# Visible Light-Induced Metal Free Surface Initiated Atom Transfer Radical Polymerization of Methyl Methacrylate on SBA-15

**DOI:** 10.3390/polym9020058

**Published:** 2017-02-10

**Authors:** Liang Ma, Na Li, Jian Zhu, Xiaodong Chen

**Affiliations:** 1 College of Chemistry, Chemical Engineering and Materials Science, Soochow University, Suzhou 215123, China; lma49@stu.suda.edu.cn (L.M.); xdchen@mail.suda.edu.cn (X.C.); 2 Jiangsu Key Laboratory of Advanced Functional Polymer Design and Application, State and Local Joint Engineering Laboratory for Novel Functional Polymeric Materials, College of Chemistry, Chemical Engineering and Materials Science, Soochow University, Suzhou 215123, China; chemzhujian@suda.edu.cn

**Keywords:** polymeric composite, surface initiated atom transfer radical polymerization, photo-induced, living radical polymerization, metal-free atom transfer radical polymerization

## Abstract

Surface-initiated atom transfer radical polymerization (SI-ATRP) is one of the most versatile techniques to modify the surface properties of materials. Recent developed metal-free SI-ATRP makes such techniques more widely applicable. Herein photo-induced metal-free SI-ATRP of methacrylates, such as methyl methacrylate, *N*-isopropanyl acrylamide, and *N*,*N*-dimethylaminoethyl methacrylate, on the surface of SBA-15 was reported to fabricate organic-inorganic hybrid materials. A SBA-15-based polymeric composite with an adjustable graft ratio was obtained. The structure evolution during the SI-ATRP modification of SBA-15 was monitored and verified by FT-IR, XPS, TGA, BET, and TEM. The obtained polymeric composite showed enhanced adsorption ability for the model compound toluene in aqueous conditions. This procedure provides a low-cost, readily available, and easy modification method to synthesize polymeric composites without the contamination of metal.

## 1. Introduction

Organic-inorganic composites based on the functionalization of mesoporous silica materials such as MCM-n, HMS-n, and SBA-n have attracted a great deal of research interest in the past decades [1,2,3,4,5,6]. Among them, SBA-15 was one of the most widely used ordered mesoporous materials for such functionalization due to its many attractive properties, such as high hydrothermal stability, desired morphology, adjustable pore sizes (2–30 nm), and thick walls. At the same time, the existence of many reactive groups on the surface, such as the –OH group, made it easy for functionalization through silanol chemistry [7,8]. Furthermore, a polymer with versatile organic groups was considered as the most efficient way to introduce different functional groups on the surface of such silica materials. Through this method, the surface properties of these silica materials could be easily tailored by changing the type and amount of polymers, which evidently enriched the functional modification of these materials. 

To covalently attach polymer chains on the surface of materials, the technique of so-called “grafting from” based on surface-initiated polymerizations is considered one of the powerful tools [9,10]. Significant advances in this area have been achieved by the development of living radical polymerization techniques, especially nitroxide-mediated polymerization (NMP) [11,12], reversible addition-fragmentation chain transfer polymerization (RAFT) [13], and atom transfer radical polymerization (ATRP) [14]. Among them, surface-initiated atom transfer radical polymerization (SI-ATRP) has received much attention during the past two decades [2,15,16,17,18]. So far, polymers with controlled structures and functional side groups can be grafted on various surfaces by SI-ATRP, as done in antifouling coatings [19], drug delivery [20], stimuli-responsive materials [21], and nanoporous membranes [22]. 

Originally, ATRP [23,24,25,26,27] was carried out with relatively high concentrations of transition metals, typically a Cu-based catalyst, in order to compensate for unavoidable radical termination reactions. Recently, several systems were developed that enabled ATRP to proceed at a catalyst loading of only 10–100 ppm of Cu [28,29]. This occurs in the presence of various reducing agents that continuously regenerate Cu^+^ activators from Cu^2+^ deactivators and it compensates for radical termination. Although catalyst loadings can be decreased to parts per million (ppm), for a variety of applications, such as microelectronics, biomaterials, etc., a key limiting factor in using ATRP is metal contamination. Very recently, Hawker et al. [30] reported a photo-induced metal-free ATRP of methyl methacrylate using 10-phenylphenothiazine (PTH) as an organic photocatalyst. In this photo-induced, metal-free ATRP mechanism, a three-component photoredox cycle is conducted. The photoexcited PTH* activates an alkyl halide and generates radicals, while the PTH^+^•Br^−^ specie deactivates the radical and regenerates the ground-state PTH. Recently, the metal-free ATRP system has been developed very fast. Various catalyst systems with improved control abilities were developed in these years [31,32,33]. With these developments, metal-free SI-ATRP also has been verified in recent years. However, these reports were focused on the modification of the surface properties of flat surfaces and particles by SI-ATRP [34,35]. Few examples of metal-free SI-ATRP on the surface of mesoporous material were reported.

Herein, for the demonstration of photo-induced metal-free SI-ATRP on mesoporous silica materials, we report a procedure to synthesize organic-inorganic hybrid materials based on photo-induced metal-free SI-ATRP of methacrylate monomers on the surface of SBA-15. Such a process was realized by chemically binding the ATRP initiator on the mesostructure walls beforehand, following by photo-induced metal-free SI-ATRP to grow polymers directly from and over the SBA-15 internal surface. Initially, methyl methacrylate was selected as the model monomer for such modification. Then, functional monomers, e.g., dimethylaminoethyl methacrylate and *N*-isopropylacrylamide, were used for a similar modification. This procedure provides a low cost, ready availability, and easy modification method to synthesize polymeric composites without the contamination of metal.

## 2. Materials and Characterization

### 2.1. Materials

The (3-aminopropyl)triethoxysilane (APTES) was purchased from Shanghai MACKLIN Reagent Co., Ltd. (Shanghai, China). and used as received. Methyl methacrylate (MMA) and dimethylaminoethyl methacrylate (DMAEMA) (Shanghai Chemical Reagents Co. Ltd., Shanghai, China) were purified before use by passing through a column filled with neutral aluminum oxide. *N*-isopropylacrylamide (NIPAM) (Shanghai Chemical Reagents Co. Ltd., Shanghai, China) was purified by recrystallization. Triethylamine (TEA, Chinasun Specialty Products Co. Ltd., Changshu, China) was dried with 4 Å molecular sieves and distilled before use. Pluronic 123 was purchased from Sigma-Aldrich (Shanghai, China) Co., Ltd. and used as received. Tetraethlorthosilicate (TEOS), hydrochloric acid (HCl), 2-bromoisobutyl bromide (BMBP), were also purchased from Shanghai Chemical Reagents Co., Ltd. and used as received. Solvents, dimethylformamide (DMF) and tetrahydrofuran (THF) were purchased from Shanghai Chemical Reagents Co., Ltd. and purified by standard methods.

### 2.2. Synthesis of SBA-15

SBA-15 was synthesized according to the procedure reported by Zhao et al. [7] using Pluronic 123 triblock copolymer as a template. Briefly, 20 g of Pluronic 123 was dissolved under stirring in 600 mL of 2 M HCl and 150 mL of deionized water at 40 °C. Then 42.5 g of tetraethlorthosilicate (TEOS) was [8] added. The resultant solution was stirred for 24 h at 40 °C before transferring into a Teflon bottle sealed in an autoclave, which was then heated to 130 °C for 24 h in an oven. The solid product was recovered by filtration and dried at 40 °C for 5 h in the vacuum oven. The template was removed from the as-made mesoporous material by calcination at 550 °C for 5 h (heating rate is 1.5 °C/min). 

### 2.3. Synthesis of SBA-APTES

Amount of 8.0 g calcined SBA-15 was degassed under vacuum at 40 °C overnight before added into a three-necked flask containing 350 mL of dry toluene and 8 mL of (3-aminopropyl)triethoxysilane (APTES). The mixture was stirred for 5 h under reflux at a nitrogen atmosphere. Under this condition, the hydroxyl groups of the SBA-15 surface react with the ethoxy groups of the APTES molecules, resulting an amino-functionalized SBA-APTES. Then, the solid was recovered by filtration and intensively washed with toluene before dried under vacuum at 40 °C overnight.

### 2.4. Synthesis of SBA-Br

Compound 2-bromo-2-methylpopionyl bromide (BMPB) was used to react with the previously attached aminopropyl groups leading the ATRP initiator bonded on SBA-15 pores surface. In this case, 8.6 g of the functionalized SBA-15 material was added to a three-necked flask containing 300 mL of dry toluene and 9 mL triethylamine. Then, 8 mL of BMPB was added in a constant pressure funnel. The system was stirred for 3 h under reflux and a nitrogen atmosphere. Finally, the solid was recovered by filtration, washed with deionized water until the filtrate was clear, and outgassed under vacuum at 40 °C overnight.

### 2.5. Synthesis of SBA-PMMA

A typical experimental procedure for the preparation of SBA-PMMA by metal-free photo-induced SI-ATRP follows: 1mL of methyl methacrylate (MMA, 0.9440 g, 9.43 mmol, 100 equiv.), 18.4 mg of ethyl 2-bromoisobutyrate (EBiB, 0.09 mmol, 1 equiv.), 5.0 mg of 10-phenylphenothiazine (PTH, 0.02 mmol, 0.2 equiv.), 1.0 g of SBA-Br, and 1 mL of DMF were added to an ampoule. The ampoule was tightly sealed and oxygen was removed by three freeze-pump-thaw cycles. The reaction was irradiated under a 3.0 mW/cm^2^ xenon lamp with the 380 nm optical filter. After a predetermined time, the ampoule was removed from the irradiation and the reaction mixture was then diluted with THF and centrifuged (10,000 rpm, 10 min) to collect the polymer-grafted SBA-15. The centrifugation and redispersion was repeated three times. The number-average molecular weight *M*_n_ and dispersity (*M*_w_/*M*_n_) were obtained by GPC using linear PMMA standards in THF as the eluent. The graft density was calculated gravimetrically. The obtained samples were denoted as SBA-PMMA.

### 2.6. Batch Adsorption

The liquid phase adsorption was ultrasonicated for 30 min and stirred for 2 h in 40 mL glass vails filled with 0.02 g of adsorbent and 10 mL of adsorbate solution which contains toluene in water with a concentration of 47 ppm. After the desired time was reached, the mixture was filtered by a nylon membrane filter (0.22 μm), then the mixture was analysed by GC. The concentration of adsorbate $C_{e}$ was calculated as the formulation followed:

(1) $$C_{e}=\frac{C_{0}}{\frac{A_{0}}{A_{e}}}$$

where $C_{0}$ is the concentration of the initial adsorbate solution; $A_{0}$ and $A_{e}$ are the GC areas of initial adsorbate solution and treated solution, respectively.

### 2.7. Characterization

Fourier transform infrared spectroscopy (FT-IR) spectra were recorded on a TENSOR 27, BRUKER Optik GmbH, Ettlingen, Germany. TGA was carried out on PerkinElmer PYRIS 1 TGA thermogravimetric analyser (PerkinElmer, Hong Kong, China) at a heating rate of 10 °C·min^−^^1^ from room temperature to 700 °C in a nitrogen atmosphere. Surface compositions were determined by X-ray photoelectron spectroscopy (XPS) on a KRA70S AXIS Ultra DLD spectrometer (Kratos Analytical Limited, Manchester, UK) at a pressure of ≈2 × 10^−^^8^ Torr using Al Kr radiation as the exciting source; the instrument was operated at 15 kV and 10 mA. The surface area was determined via the nitrogen adsorption/desorption technique at 77 K using the ASAP 2020 surface area and porosimetry analyzer. The standard BET and DFT models were applied to determine the surface area and pore volume. The number-average molecular weight (*M*_n,GPC_) and molecular weight distribution (*Ð*) of the polymers were determined by a TOSOH HLC-8320 equipped with a refractive-index detector, using a TSKgel guard column SuperMP-N (4.6 mm × 20 mm) and two TSKgel Supermultipore HZ-N (4.6 mm × 150 mm) with a measurable molecular weight ranging from 5 × 10^2^ to 5 × 10^5^ g/mol. DMF (+LiBr 0.1% weight) was used as the eluent at a flow rate of 0.6 mL/min and 40 °C. GPC samples were injected using a TOSOH plus auto sampler and calibrated with PS standards purchased from TOSOH (Tokyo, Japan). The concentration of toluene in the treated solution was quantitatively analyzed using a GC2010 (Shimadzu, Kyoto, Japan) plus gas chromatography with a low-polarity capillary column and a flame ionization detector (FID). The oven temperature was initially set at 80 °C, and held this temperature for 5 min, then ramped at 5 °C·min^−^^1^ to 140 °C, and held at this temperature for 2 min. The temperatures of injector and detector were at 280 and 300 °C respectively.

## 3. Results

**Scheme 1:**
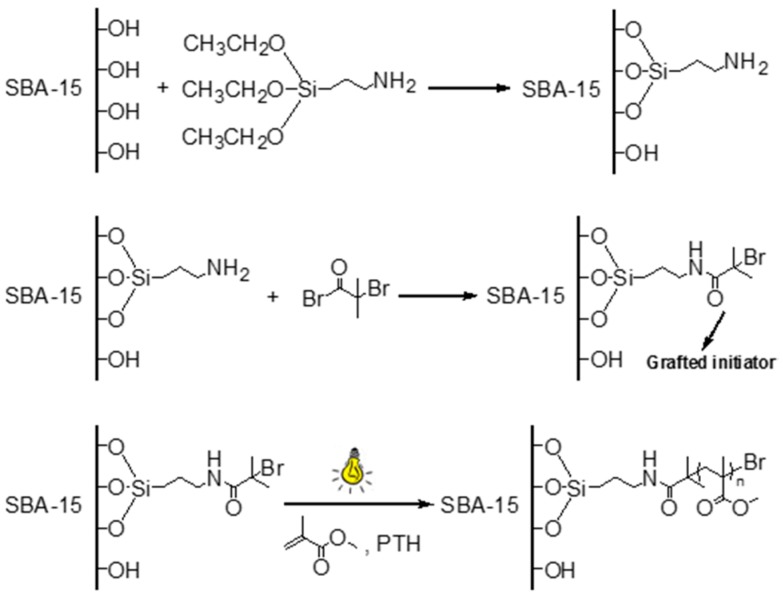
Synthetic procedure of SBA-PMMA.

**Figure 1:**
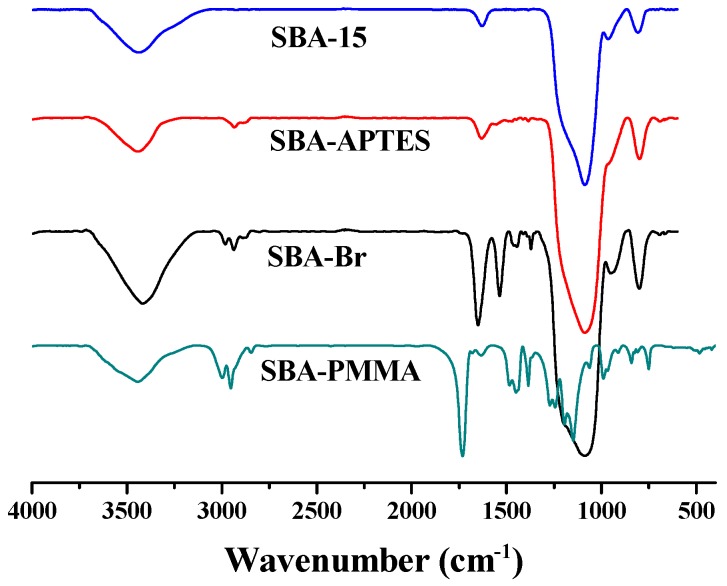
FT-IR spectra of SBA-15, SBA-APTES, SBA-Br, and SBA-PMMA.

SI-ATRP using functionalized SBA-15 as the initiator was carried out under visible-light irradiation with the presence of PTH. The synthetic route is shown in Scheme 1, and it was similar to the literature [3,30]. Thus, in order to carry out SI-ATRP on the surface of SBA-15, the initiator moiety was firstly anchored onto the surface of the material. Then, the photo-induced SI-ATRP was carried out. The FT-IR was used to monitor the structure evolution during the processing. The results are shown in Figure 1. Pristine SBA-15 showed a strong peak at 3440 cm^−1^, corresponding to Si–OH stretching vibrations. After the introduction of the initiator, peaks corresponding to C–H stretching vibrations at 2980 and 2920 cm^−1^, –NH–CO– vibrations at 1535 cm^−1^, and C–Br vibrations at 800 cm^−1^ were observed in the spectrum of SBA-Br. Such results implied the successful introduction of the ATRP initiator functional moiety onto the surface of SBA-15 by silanol chemistry, followed by the amidation reaction. Furthermore, the strong peak at 1720 cm^−1^ according to –C=O vibrations in PMMA could be found in the spectrum of SBA-PMMA, which indicated the attachment of the PMMA chain on the surface of SBA-15.

**Figure 2:**
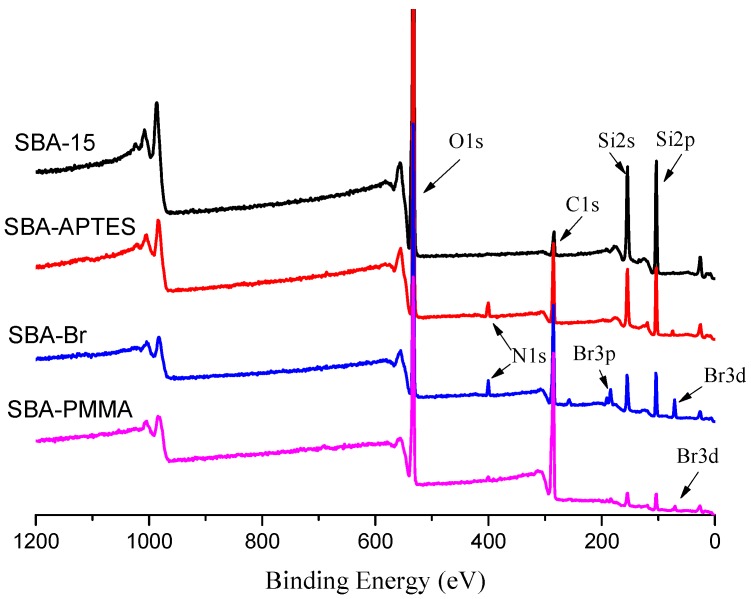
XPS spectra of SBA-15, SBA-APTES, SBA-Br, and SBA-PMMA.

To further confirm the surface structure, the materials obtained at different stages were characterized by XPS. The wide scan spectra of SBA-15, SBA-APTES, SBA-Br, and SBA-PMMA are summarized in Figure 2. It shows that the signal according to nitrogen was observed after SBA-15 was treated with 3-aminopropyltriethoxysilane (APTES). Such a result indicated that the APTES successfully anchored onto the surface of SBA-15. The signals according to the nitrogen and bromine atoms were found in the XPS survey after SBA-APTES was treated with 2-bromoisobutyryl bromide (BMPB), which implied the successful reaction between the surface amine groups with BMPB. Combined with the results of the FT-IR, it clearly showed the successful introduction of ATRP initiating groups onto the surface of SBA-15. Furthermore, after the SI-ATRP of methyl methacrylate (MMA), the signals of nitrogen and bromine still remained in the spectrum, while the intensity weakened, which implied the successful surface-initiated polymerization.

After these initial surveys, the polymerizations of different monomers, e.g., MMA, DMAEMA, and NIPAM, were carried out using SBA-Br as the initiator, ethyl 2-bromoisobutyrate (EBiB) as the co-initiator and PHT as the photocatalyst under the irradiation of a xenon lamp with a 380 nm optical filter at 30 °C. The light intensity was 3.0 mW/cm^2^. The polymerization results are summarized in Table 1. The molecular weight of the PMMA obtained in the solution was measured by GPC using THF as the eluent and calibrated by PMMA standards. The molecular weights of the DMAEMA and NIPAM were measured by GPC using DMF as the eluent and calibrated by PMMA standards. It showed that non-polymerization took place without the light irradiation or PTH after 72 h at 30 °C (Entries 1 and 2 in Table 1). The polymerization could be carried out smoothly after adding PTH as the photocatalyst under light irradiation (Entries 3–9 in Table 1). A monomer conversion as high as 57.6% was obtained after 72 h of polymerization (Entry 7 in Table 1). The grafting ratio of the polymer reached as high as 27.6%, which was similar to the results in the literature [36]. Not only could the monomer MMA be grafted onto the surface of SBA-15, but also the monomers PDMAEMA and PNIPAM could be grafted onto the surface of SBA-15 (Entries 8 and 9 in Table 1), which implies the various applications for the current method for grafting polymers from the surface of SBA-15. The polymerization showed controlled characteristics, e.g., controllable molecular weights along with a narrow molecular weight distribution of the obtained polymers. 

**Figure 3:**
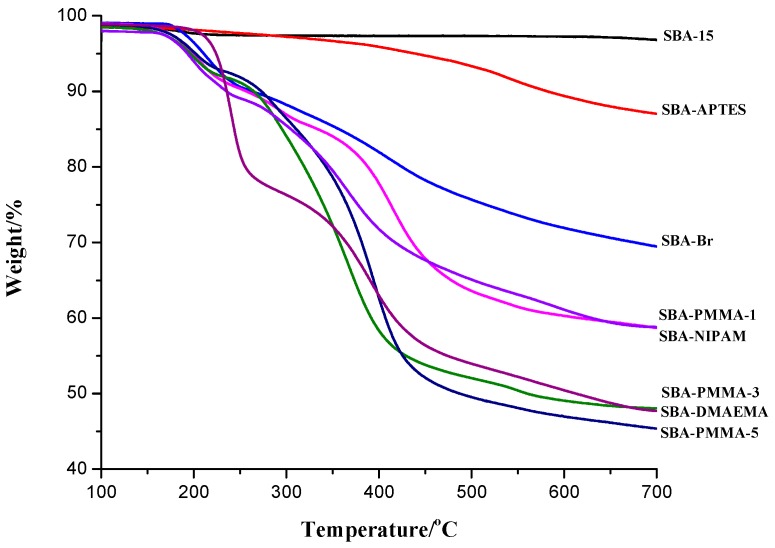
TGA curves of SBA-15; SBA-APTES; SBA-Br; SBA-PMMA; SBA-DMAEMA; and SBA-NIPAM. TGA was performed under the protection of N_2_ at a heating rate of 10 °C·min^−1^.

The photo-induced metal-free SI-ATRPs on the surface of SBA-15 of different monomers were also monitored by TGA. The TGA curves of SBA-15, SBA-APTES, SBA-Br, and SBA-PMMA at different conversions, SBA-DMEAME, and SBA-NIPAM were showed in Figure 3. The polymer chains started to decompose at 250 °C in a nitrogen atmosphere due to the elimination of the ester group in the polymer chains. It showed that the amount of weight loss increased with the polymerization time. Such results implied that the amount of polymer grafted on SBA-15 increased with the polymerization time. The grafting percentages of the polymer on the surface of SBA-15 could be calculated from the TGA data, and are shown in Table 1. The grafting ratio varied in the range of 12.5%~27.6% by changing the polymerization time, which offered a convenient way to adjust the amount of polymers on the surface of SBA-15. The grafting ratio of the current system was slightly lower than the results reported in the literature, which may have been caused by the porous structure of SBA-15 [37].

**Figure 4:**
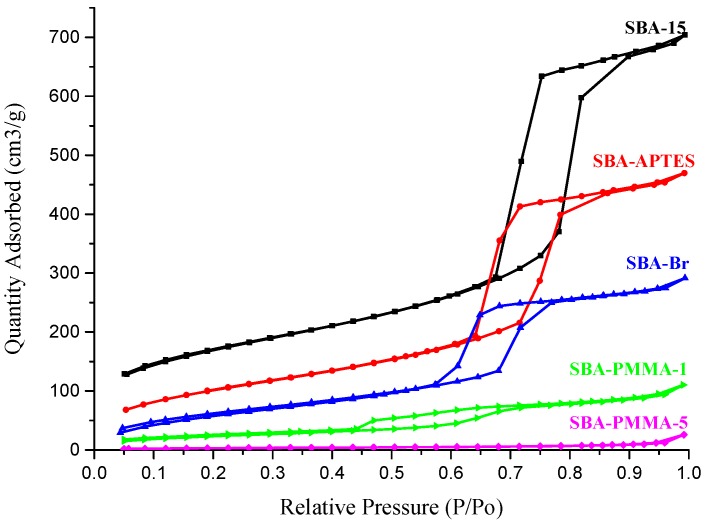
N_2_ adsorption-desorption isotherms of SBA-15; SBA-APTES; SBA-Br; SBA-PMMA-1; and SBA-PMMA-5.

One of important properties of mesoporous materials is their porous structure. Thus, in order to investigate the effect of surface grafting on the porous structure, the N_2_ adsorption-desorption isotherms of polymer-grafted SBA-15 materials together with the pure-silica SBA-15 sample were characterized. The results are shown in Figure 4. The BET surface area (*S*_BET_) and total pore volume (*V*_total_) are given in Table 2. The pure-silica SBA-15 sample displayed a type IV isotherm with H1 hysteresis and a sharp increase in volume adsorbed at *P*/*P*_0_ ≈ 0.78 with a pore volume of 1.09 cm^3^/g, a characteristic of highly ordered mesoporous materials. For samples SBA-APTES, SBA-Br, and SBA-PMMA-1, they all exhibited a type IV isotherm with a H1 hysteresis loop with a lower specific area and a slightly smaller pore volume in comparison with SBA-15, e.g., 0.73, 0.45, and 0.17 cm^3^/g, respectively. However, with increasing the amount of PMMA from the grafting ratio of 12.5% in SBA-PMMA-1 to 27.6% in SBA-PMMA-5 on the surface of SBA-15, the shape of curve was changed with a pore volume of only 0.04 cm^3^/g. The surface area also decreased dramatically after the introduction of the PMMA polymer chain, e.g., from 594.4 m^2^/g of prism SBA-15 to 86.8 and 11.4 m^2^/g of SBA-PMMA-1 and SBA-PMMA-5. The above physisorption data indicated that, in the presence of a relatively low grafted density, the textural properties of SBA-15 were substantially maintained. The pore volume was decreased with the increasing amount of introduced PMMA, which was due to the polymer occupying the pore volume.

**Figure 5:**
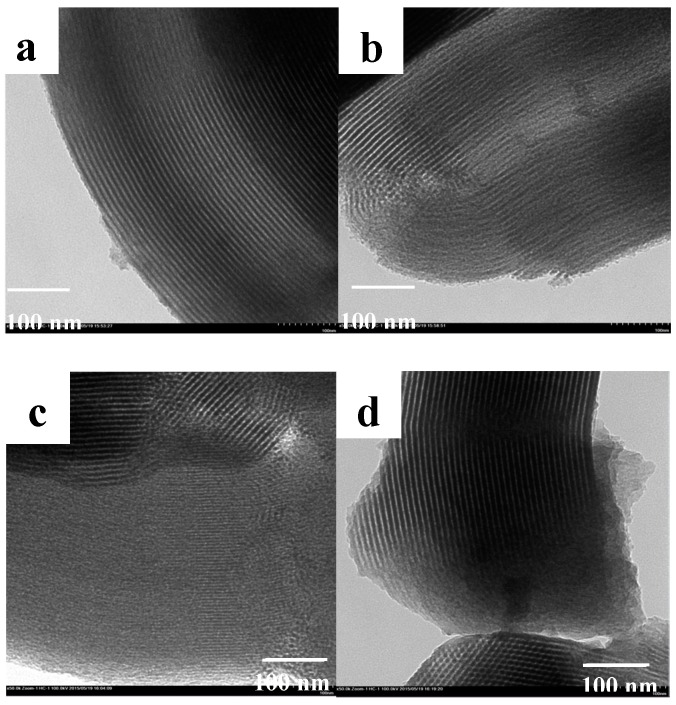
TEM images of (**a**) pure-silica SBA-15; (**b**) SBA-APTES; (**c**) SBA-Br; and (**d**) SBA-PMMA-5.

The occupation of the polymer in the pores of SBA-15 after polymerization was verified by the TEM images before and after the polymerization. The TEM images of the pure-silica SBA-15, SBA-APTES, SBA-Br, and SBA-PMMA-5 are compared in Figure 5. The ordered, arranged pore arrays of the pure-silica SBA-15 could be clearly seen (Figure 5a). Such ordered pore arrays were gradually disrupted after the introduction of APTES and Br onto the surface of SBA-15. The situation was obvious after the introduction of PMMA onto the surface. However, most of the ordered structure could be kept by controlling the amount of introduced polymer, which was easy to realize by using the SI-ATRP technique. These results agreed well with the results observed in the BET characterization, which showed the low BET surface area of SBA-PMMA with a high grafted density.

**Figure 6:**
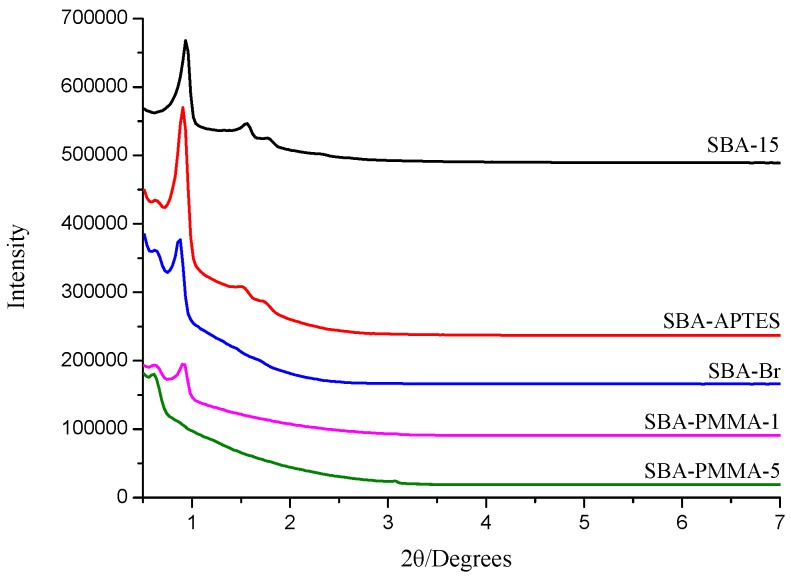
The small and large angles of powder XRD patterns of SBA-15, SBA-APTES, SBA-Br, SBA-PMMA-1 and SBA-PMMA-5.

The effect of such modification on the ordered structure of SBA-15 was further monitored by XRD characterization. Figure 6 shows the powder XRD patterns of pure-silica SBA-15, SBA-APTES, SBA-Br, and PMMA-grafted SBA-15 samples. It shows that the pure-silica SBA-15 exhibited three well-resolved XRD peaks in the region of 2θ = 0.5°–2.0°, which can be indexed to the (100), (110), and (200) diffractions. The peak positions for the samples remained constant after the amine-functionalization process, suggesting high stability of the materials. However, after treating with BMPB and grafting with PMMA, a decrease in the diffraction peak intensity was observed, indicating the decrease of crystallinity in the materials. These peaks even disappeared after introducing a large amount of PMMA on the surface of SBA-15. Combining the results obtained from BET, TEM and XRD, the textural properties of SBA-15 could be changed from an ordered structure to a disordered structure after introducing different amounts of polymer. It was important to control the amount of polymer introduced onto the surface of SBA-15 for maintaining the ordered structure of SBA-15. 

**Figure 7:**
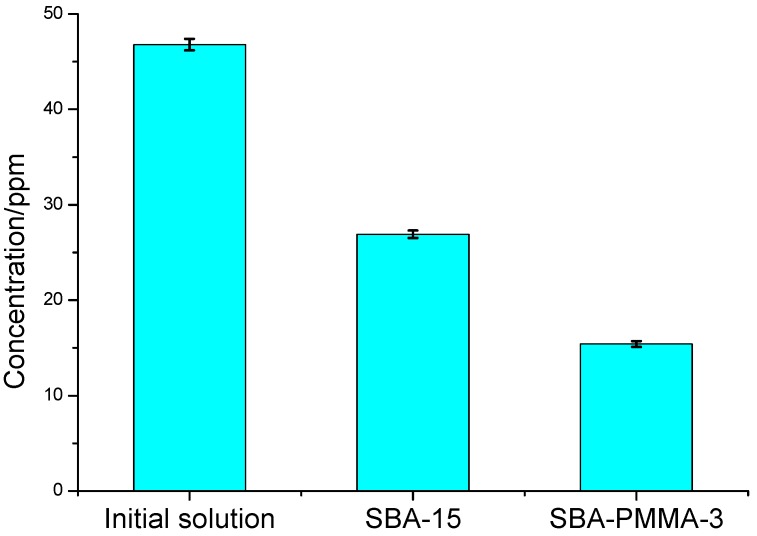
Adsorption abilities of SBA-15 before and after the polymer modification. Conditions: 10 mL of toluene in water solution with concentration of 47 ppm was added with 20 mg adsorbent. The mixture was stirred 24 h at ambient temperature.

SBA-15 has been widely applied in adsorption materials due to its huge surface area and mesoporous structure. Herein, the adsorption properties of SBA-15 before and after modification were investigated. Toluene was used as the model adsorbate and aqueous containing 47 ppm of toluene was used as model solution for the adsorption investigation. The results are summarized in Figure 7. It shows that 26.9 ppm of toluene remained in the solution after the adsorption by pristine SBA-15. The adsorption ability could be improved after using PMMA-modified SBA-15, e.g., there was 15.4 ppm of toluene remaining in the solution after using PMMA-modified SBA-15 as the adsorbent. Thus, the adsorption properties of SBA-15 could be enhanced by attaching a polymer onto the surface.

## 4. Conclusions

The metal-free photo-induced SI-ATRP of methacrylate on the surface of mesoporous SBA-15 was demonstrated. The polymerization conditions were optimized, and using this metal-free photo-induced SI-ATRP, a SBA-15-based polymeric composite with an adjustable graft density and grafted polymer chain length was obtained. It showed that the porous structure could be modified over a large range after the introduction of the polymer chain. Enhanced adsorption ability for toluene was obtained after modifying SBA-15 with PMMA. The use of photo-initiation is ideal as it avoids the introduction of metals as catalysts which can remain behind as impurities. This procedure provides a low-cost, readily available, and easy modification method to synthesize polymeric composites without the contamination of metals with enhanced adsorption ability. 

## Figures and Tables

**Table 1:** Results of photo-induced metal-free SI-ATRP of MMA using SBA-Br as an initiator ^a^.

Entry	Label	Time (h)	Conv. (%)	*M*_n,GPC_^d,e^ (g/mol)	*Ð* ^d,e^	Grafting Ratio ^f^ (%)
1	SBA-PMMA-C1 ^b^	72.0	--	--	--	--
2	SBA-PMMA-C2 ^c^	72.0	--	--	--	--
3	SBA-PMMA-1	15.0	10.8	12,800	1.24	12.5
4	SBA-PMMA-2	24.0	9.4	11,100	1.29	21.6
5	SBA-PMMA-3	36.0	20.0	13,300	1.28	24.6
6	SBA-PMMA-4	48.0	20.7	12,800	1.24	25.0
7	SBA-PMMA-5	72.0	57.6	16,700	1.27	27.6
8	SBA-DMAEMA ^e^	20.0	83.4	21,100	1.83	23.8
9	SBA-NIPAM ^e^	18.0	85.0	13,400	2.25	14.4

^a^ [monomer]_0_/[EBiB]_0_/[PTH]_0_ = 100/1/0.2; SBA-Br = 0.1 g. Polymerized at 30 °C; ^b^ Without UV irradiation. [monomer]_0_/[EBiB]_0_/[PTH]_0_ = 100/1/0.2; SBA-Br = 0.1 g; ^c^ With UV irradiation. [monomer]_0_/[EBiB]_0_/[PTH]_0_ = 100/1/0; SBA-Br = 0.1 g; ^d,e^ Molecular weight of free polymers obtained from the solution determined by GPC using narrow poly(methyl methacrylate) standards, THF as the eluent. The molecular weight of PDMAEMA and PNIPAM were determined by GPC using narrow poly(methyl methacrylate) standards, DMF as the eluent; ^f^ The grafting ratio was referred to the weight percentage of polymer to SBA-15 which was measured by TGA.

**Table 2:** Results of BET surface area and total pore volume of SBA-15, SBA-APTES, SBA-Br, and SBA-PMMA.

Entry	BET Surface Area (m^2^/g)	Pore Volume (cm^3^/g)	Pore Size (nm)	Grafting Ratio (%)
SBA-15	594.4	1.09	7.33	-
SBA-APTES	378.3	0.73	7.68	-
SBA-Br	239.3	0.45	7.54	-
SBA-PMMA-1	86.8	0.17	7.86	12.5
SBA-PMMA-5	11.4	0.04	14.0	27.6

## Supplementary Materials

The following are available online at www.mdpi.com/2073-4360/9/2/58/s1. Figure S1: Polymerization kinetics of MMA using SBA-Br as the initiator and EBiB as the co-initiator under the conditions of [monomer]0/[EBiB]0/[PTH]0 = 100/1/0.2; SBA-Br = 0.1 g. Polymerized at 30 °C; Figure S2: Evolution of molecular weight and molecular weight distribution of PMMA with conversion using SBA-Br as the initiator and EBiB as the co-initiator under the conditions of [monomer]0/[EBiB]0/[PTH]0 = 100/1/0.2; SBA-Br = 0.1 g. Polymerized at 30 °C; Figure S3: GPC traces of PMMA obtained in the polymerization using SBA-Br as the initiator and EBiB as the co-initiator under the conditions of [monomer]0/[EBiB]0/[PTH]0 = 100/1/0.2; SBA-Br = 0.1 g. Polymerized at 30 °C; Figure S4: 1H-NMR spectrum of SBA-PMMA-5 obtained by using SBA-Br as the initiator and EBiB as the co-initiator under the conditions of [monomer]0/[EBiB]0/[PTH]0 = 100/1/0.2; SBA-Br = 0.1 g. Polymerized at 30 °C.
