# Supplementary material for: Visible Light-Induced Metal Free Surface Initiated Atom Transfer Radical Polymerization of Methyl Methacrylate on SBA-15
Source: Polymers (Basel). 2017 Feb 10;9(2):58. doi: 10.3390/polym9020058 (PMC6432016; doi:10.3390/polym9020058)
Supplement: Supplementary file 1 [file polymers-09-00058-s001.pdf]

Supplementary Materials: Visible Light-Induced Metal Free Surface Initiated Atom Transfer Radical Polymerization of Methyl Methacrylate on SBA-15

Liang Ma, Na Li, Jian Zhu and Xiaodong Chen

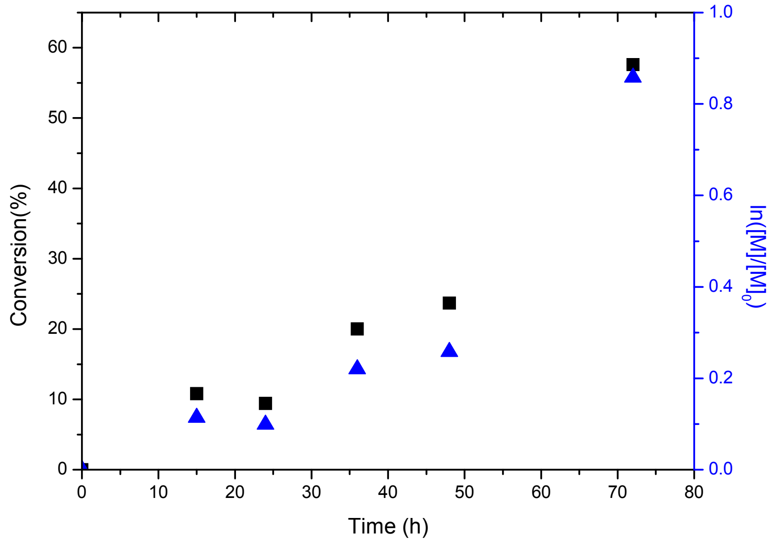

Figure S1. Polymerization kinetics of MMA using SBA-Br as the initiator and EBiB as the co-initiator under the conditions of $[monomer]_0/[EBiB]_0/[PTH]_0$ = 100/1/0.2; SBA-Br = 0.1 g. Polymerized at 30 °C.

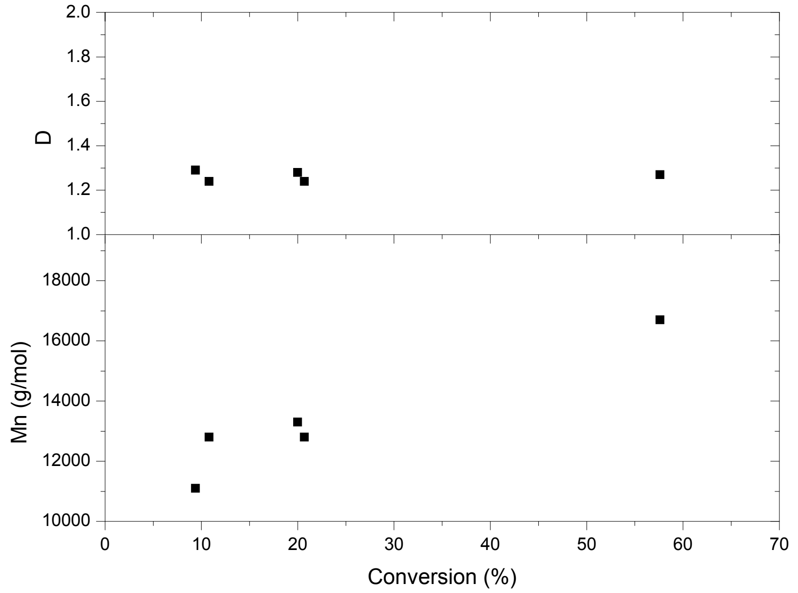

Figure S2. Evolution of molecular weight and molecular weight distribution of PMMA with conversion using SBA-Br as the initiator and EBiB as the co-initiator under the conditions of $[monomer]_0/[EBiB]_0/[PTH]_0$ = 100/1/0.2; SBA-Br = 0.1 g. Polymerized at 30 °C.

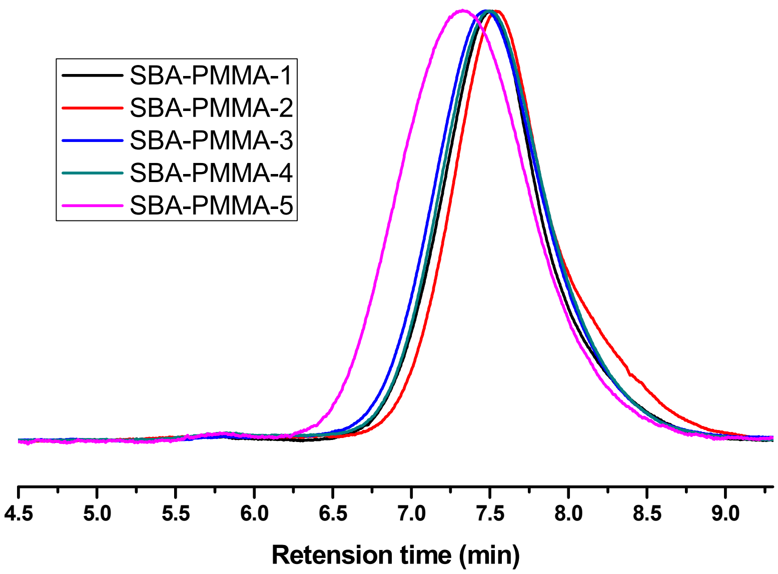

Figure S3. GPC traces of PMMA obtained in the polymerization using SBA-Br as the initiator and EBiB as the co-initiator under the conditions of $[monomer]_0/[EBiB]_0/[PTH]_0$ = 100/1/0.2; SBA-Br = 0.1 g. Polymerized at 30 °C.

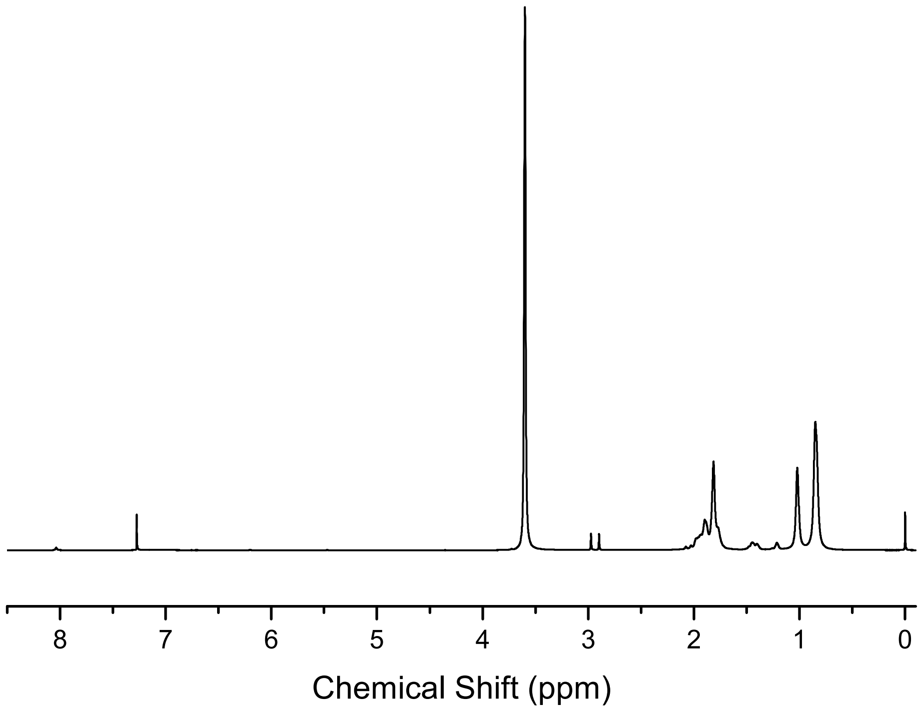

Figure S4. ^{1}H-NMR spectrum of SBA-PMMA-5 obtained by using SBA-Br as the initiator and EBiB as the co-initiator under the conditions of $[monomer]_0/[EBiB]_0/[PTH]_0$ = 100/1/0.2; SBA-Br = 0.1 g. Polymerized at 30 °C.
